# Practical Use of the X-Ray in Dentistry

**Published:** 1903-04

**Authors:** L. E. Custer

**Affiliations:** Dayton, Ohio


### PRACTICAL USE OF THE X-RAY IN DENTISTRY.[1]

BY DR. L. E. CUSTER, DAYTON, OHIO.

In presenting this subject this evening, in the presence of those most expert as well as of those who have not given this subject much attention, I will try to strike a medium and see if it is not possible to make an entertaining and instructive evening.

In order to explain the nature of the X-ray, I have placed a chart upon the wall which will show a comparison between the air and ether vibrations. Air is valuable chiefly to us for the oxygenation of the blood and for hearing. From the top of a high mountain we suffer from lack of air, as shown by rapid breathing, and we also experience difficulty in hearing at a great distance. Sound, as you will see by the chart, is produced by exceedingly low air vibrations. On the other hand, we have the ether vibrations, and it is through them that we derive electric phenomena,

---

[1] Read before the Academy of Stomatology, Philadelphia, October 28, 1902.

and it is by reason of them that the X-ray and wireless telegraphy are possible. It is a matter of coincidence that the two phenomena, the X-ray and Hertz waves, have been demonstrated in recent years. It is through the ether that we obtain light, heat, and all electric phenomena. It is a material which fills all space.

The range of human sight is one which reaches, so far as we know, from four hundred billions to seven hundred and fifty billions of ether vibrations. Beyond that we have the actinic rays, and beyond that the X-rays; at the other extreme we have the infra-red and the Hertz waves.

The discovery of the X-ray was made by Röntgen in 1895, partly as the result of an accident. Up to this time, however, others had been at work along the same line. The Geissler tube had been in use for years, and is a tube of low vacuum. This shows how under certain conditions an electric charge will pass through a partial vacuum more easily than through the air. Some ten years after the invention of the Geissler tube came the Crookes tube. This is one of exceedingly high vacuum from which nearly all the air has been exhausted.

The ray is sent off from the cathode, and, striking the anode at an angle of forty-five degrees, is reflected therefrom at such an angle that it can be used. The X-ray differs from the Leonard ray in that it cannot be deflected by a magnet or refracted; also, in that it passes out through the tube without any perceptible interference.

I have in my hand a screen of barium-platino-cyanide, and this becomes fluorescent when the rays pass through it. When I pass my arm before it the rays are stopped by the bone and also to some extent by the flesh, and that gives the distinction between the bone and the flesh. I will put on another tube and you will see a little difference in the effect of a different degree of vacuum. An important point in radiography is the condition of the tube as to vacuum, for upon this depends the appearance of the finished picture. Taking the hand as an illustration, we have about the conditions with which the dentist has to deal. A tube of low vacuum is one which will give a picture of greater differentiation between the bone and the flesh or between the tooth and the alveoli. In operating upon the jaw with a tube of comparatively high vacuum we would not get much contrast between the teeth and the bone with which they are surrounded.

The method by which the X-ray is generated, or the means by which we get this tremendous pressure of current are three,—the Tesla coil, not used to any extent; the static machine, and the plain induction coil. The majority of those engaged in X-ray work alone are using the induction coil, while physicians, as a rule, are using the static machine, because for their purposes it has other values. I should recommend the use of the coil to the dentists. The induction coil as shown here consists of a primary coil, and surrounding that is a secondary winding. The primary is a coil of wire of about two layers covering a bundle of soft iron wire. This is independent of the secondary coil. There are fifteen miles of wire in the secondary coil. This has no metallic connection with the primary; on the contrary, the greatest care is used in insulation of the secondary from the primary. We have not only a half-inch thickness of ebonite, but a thickness of paraffin, beeswax, and resin.

The efficiency of the coil depends, I think, largely upon the interrupter. Not until I had used nearly every interrupter upon the market did I finally decide upon a form of the Caldwell interrupter as the best. By the diagram on the board you will see that not only the perfection of its operation is a good feature, but also the simplicity of it. You will find it gives perfect work under all conditions. It consists of a glass jar in which is a strip of lead and within that a porcelain vessel of almost any kind perforated near the bottom by a small opening the size of a No. 10 or 12 wire, and in which is suspended a strip of lead. The jar is filled about two-thirds with sulphuric acid and water, in the proportions of three of water to one of acid. The theory on which it interrupts the current quickly, decisively, and clearly is that the current, in order to pass from the one electrode to the other, must pass through an opening in the porcelain cup. The solution is decomposed by the current as it passes through the opening, and the moment a bubble of gas forms there is complete insulation between the two electrodes. The connection is quickly renewed by reason of the gas rising to the surface. The frequency of the interruption depends upon the thinness of the porcelain, the volume of the current, and upon the size of the opening. This has been the most successful interrupter, and I would recommend it for nearly all uses because of its simplicity and perfect performance under all conditions.

In the use of the Caldwell interrupter we do away with the use of the condenser, an expensive part of the instrument, and with the uncertainty of the operation of the vibrator. A coil which will give an eight-inch spark will fulfil all requirements. There are localities in which physicians would be very glad to avail themselves of this appliance, and you would then need a twelve- or fifteen-inch coil. Yet all the work which comes strictly under the dentist's care can be done with an eight-inch coil. The apparatus should cost scarcely more than seventy-five dollars. The tubes would cost from ten to fifteen dollars, a fluoroscope about the same, and the interrupter can be made for about a dollar. That should be the limit of the expense, and I do not see why the work should not be taken up by many. It is not expected that every dentist should take up this work, but to those who do I hope my remarks may be of benefit.

I will go through the details of taking a skiagraph or two. These films for dental purposes are about the size of a postage-stamp, and I enclose them in two layers of black paper. I will pass around some of the pictures which I am taking in this manner. The pictures comprise an alveolar abscess, the condition of a third molar at thirteen years of age, outline of an antrum, complete absorption of the posterior root of the first molar, a retained temporary molar, the absence of a third molar, and an impacted third molar.

The X-ray burn in the first days was a formidable occurrence, but at this day in the hands of dentists and under the requirements for dental work we may say that it is not necessary to produce a burn. I have never produced a burn in dental X-ray operations, and do not expect to do so. The cases which come under the hands of physicians, and especially those in which the search for calculi is made, require a condition of tube which sometimes produces a burn, but for dental purposes the matter of a burn is not to be considered. As fifteen to twenty minutes are required to produce a burn, and but three to ten seconds' exposure is necessary for dental skiagraphs, we therefore have a wide margin of safety.

The burn is somewhat similar to an ordinary burn, yet it is entirely different pathologically. It has the appearance of a scald in the third or fourth stage, and may cause sloughing.

In a therapeutic way the X-ray is being used largely and with some marked degree of success in the treatment of certain lesions, especially epithelioma, lupus, and the like. This would come under the hands of the regular practitioner, and yet, if dentists in the country or smaller towns had the X-ray appliance, they would be called upon to give the treatment.

(Answering questions.) Pulp nodules can be definitely distinguished, but it is impossible to distinguish a lateral perforation.

